# A Composite Microfiber for Biodegradable Stretchable Electronics

**DOI:** 10.3390/mi12091036

**Published:** 2021-08-28

**Authors:** Adeela Hanif, Gargi Ghosh, Montri Meeseepong, Hamna Haq Chouhdry, Atanu Bag, M. V. Chinnamani, Surjeet Kumar, Muhammad Junaid Sultan, Anupama Yadav, Nae-Eung Lee

**Affiliations:** 1 School of Advanced Materials Science and Engineering, Sungkyunkwan University (SKKU), Suwon 16419, Kyunggi-do, Korea; adeelahaneef@gmail.com (A.H.); gargisimita@gmail.com (G.G.); atanubagec@gmail.com (A.B.); chinnabme@gmail.com (M.V.C.); surjeetkhurana9@gmail.com (S.K.); mjunaidsultan30@gmail.com (M.J.S.); anupama.yadav.biotech@gmail.com (A.Y.); 2 SKKU Advanced Institute of Nanotechnology (SAINT), Sungkyunkwan University (SKKU), Suwon 16419, Kyunggi-do, Korea; chivazh.mm@gmail.com (M.M.); Hamna.haq.ch@gmail.com (H.H.C.); 3 Biomedical Institute for Convergence at SKKU (BICS), Sungkyunkwan University (SKKU), Suwon 16419, Kyunggi-do, Korea

**Keywords:** transient electronics, stretchable electronics, microfiber, poly(glycerol sebacate), poly(vinyl alcohol), biodegradable

## Abstract

Biodegradable stretchable electronics have demonstrated great potential for future applications in stretchable electronics and can be resorbed, dissolved, and disintegrated in the environment. Most biodegradable electronic devices have used flexible biodegradable materials, which have limited conformality in wearable and implantable devices. Here, we report a biodegradable, biocompatible, and stretchable composite microfiber of poly(glycerol sebacate) (PGS) and polyvinyl alcohol (PVA) for transient stretchable device applications. Compositing high-strength PVA with stretchable and biodegradable PGS with poor processability, formability, and mechanical strength overcomes the limits of pure PGS. As an application, the stretchable microfiber-based strain sensor developed by the incorporation of Au nanoparticles (AuNPs) into a composite microfiber showed stable current response under cyclic and dynamic stretching at 30% strain. The sensor also showed the ability to monitor the strain produced by tapping, bending, and stretching of the finger, knee, and esophagus. The biodegradable and stretchable composite materials of PGS with additive PVA have great potential for use in transient and environmentally friendly stretchable electronics with reduced environmental footprint.

## 1. Introduction

Recently, stretchable electronic materials that can be integrated seamlessly with deformable, dynamic, and irregular surfaces, particularly on the human body, have presented new opportunities for wearable applications [1,2,3,4,5,6]. In addition to stretchable electronics, transient electronics built with biodegradable materials are of increasing interest for future wearable and implantable applications [2,6,7,8,9,10]. Furthermore, a range of biodegradable devices could attain a high level of functionality by introducing stretchability and the thoughtful merging of soft-to-hard materials [11]. Biodegradable and stretchable electronics impart unique features to on-body or in-body bio-integrated systems [12,13]. For real-time monitoring of parameters such as pressure, strain, pH, oxygen saturation, and temperature, the transient electronics would improve the acquisition capability of time-limited information about post-surgical infections, tissue healings, and personalized treatments [14,15,16,17,18]. Biodegradable and stretchable electronic devices are anticipated for dynamic and short-term wearable health monitoring with the smallest environmental footprint. The biodegradable devices have been used as single-time point-of-care diagnostics as they reduce the waste-related with single-time-use disposable sensors and are environmentally friendly too. However, imparting stretchability to biodegradable devices remains challenging. In particular, the major hurdle is the synthesis of new biodegradable materials with high functionality, such as stretchability, biocompatibility, strength, and electrical conductivity [7,10,19,20,21,22,23].

To be well-suited for applications in transient stretchable electronics, devices need to meet various requirements, including biodegradability, biocompatibility, high stretchability, mechanical stability, and reliable response under repetitive mechanical deformation [19,21,23,24]. Therefore, the development of biodegradable materials that can maintain their structural integrity with high stretchability, adhere to biological tissues conformally, and produce less environmental waste is in great demand. An increasing number of biodegradable electronic components [25,26,27], such as resistors, transistors [10], light-emitting electrochemical cells [28,29,30], and capacitors [31] have been reported in recent years. However, they have been fabricated on foil or rigid substrates [32] that lack stretchability. Naturally existing materials, such as silk fibroin [33], gelatin [34,35], cellulose [36], and alginate, have been used in elastic materials by adding a high content of plasticizer to create a hydrogel [37]. However, the poor stretchability and brittleness of silk fibroin and cellulose films prevent the electronics from comfortably cohering to dynamic skins. Synthetic biodegradable polymers (polylactic acid) are brittle and have a high elastic modulus (around 50 MPa) [38], and poly (caprolactone) (PCL) [39], polyhydroxyalkanoates [38], and poly (1,8-octane diol-*co*-citrate) [40] are rarely stretchable. Researchers have been looking further into synthetic elastomers to achieve full control of the desired mechanical and electrical properties [37,41,42,43]. Among synthetic polymers, poly (glycerol sebacate) (PGS) is an inexpensive biodegradable elastomer. The rubber-like elasticity of PGS provides sustainability and helps to recover the elastomer from the deformation to well match with the elastic properties of soft tissues of the dynamic human body. The elasticity of PGS could allow elimination of the mismatch between the dynamic and soft tissues and mechanically stiff/rigid electronics. PGS is a body-compatible, ecofriendly, and biodegradable elastomer that can withstand the strain of ~20%, which makes it a potential candidate to be used in the stretchable and on-body wearable electronic device [15]. Initially, it was designed for soft tissue engineering due to its excellent recovery from deformation [37]. PGS elastomer is well suited for cell growth and cell adhesion. This biodegradable elastomer has been studied in a dynamic environment as a scaffold (e.g., cartilage and heart) [44]. PGS has an elastic modulus ranging from 0.25 to 1.45 MPa and a tensile strength ranging from 0.3 to 1.5 MPa [45]. The major drawback of PGS is its poor processability resulting from low solubility and formability especially after thermal crosslinking. PGS can be produced with the prepolymer, but the resulting structure melts during the thermal crosslinking step and loses its well-defined shape [46]. The mechanical properties of PGS are varied in a narrow range only and its strain at break less than 20% does not match with that of human skin, which is greater than 20%. To overcome those problems, one approach is to add a filler into the PGS matrix. Blending PGS with other polymers such as PCL [47,48], poly-l-lactic acid [49], poly (octamethylene maleate (anhydride) citrate) [50], polyaniline [51], polyurethane [52], and polyglycerol sebacate-*co*-polyethylene glycol [53], or embedding nanofillers, such as carbon nanotubes [54,55,56] and graphene [51], improved the mechanical strength, demonstrating the effectiveness of combining other polymers or nanofillers to optimize overall performance while reducing the elastic modulus and extensibility. Blending synthetic polymers would compromise biodegradability and stretchability, as some of the synthetic polymers are brittle. The trade-off between stretchability and biodegradability remains challenging when blended with highly stiff and brittle materials.

Herein, the goal of our research was to synthesize a stretchable, biocompatible, biodegradable and reinforced composite microfiber of PGS and polyvinyl alcohol (PVA) with Young’s modulus suitable for wearable applications. PVA is a stiff polymer with a high range of Young’s modulus with lower elongation at break that allows it to overcome the issues of low viscosity, difficult handling, poor formability, and time consumption during crosslinking of PGS. PVA is biodegradable in aerobic and anaerobic conditions, biocompatible, odorless, non-toxic, and has been used in biomedical research fields [57]. The incorporation of PVA polymer into PGS could enhance the mechanical properties of the PGS microfiber, making it suitable for on-body applications. The wet spinning process was used for microfiber fabrication where the polymer solution coagulates to form microfibers, and an extra step that involved the curing of PGS at high temperatures over a long curing period for making films was not required. Furthermore, the biocompatibility and the biodegradation profiles of microfibers were investigated. The recovery of microfiber after three cycles of extension demonstrated mechanical stability when subjected to load due to the elasticity imparted by PGS to the composite. Finally, a biodegradable composite-based strain sensor based on the microfiber incorporated with Au nanoparticles (AuNPs) was demonstrated as a skin-mountable device. The developed sensor showed the capability of monitoring the skin strains with a good sensibility. The biodegradable and stretchable composite of PGA and PVA has the potential as a suitable candidate for challenging applications in the field of transient stretchable electronics.

## 2. Materials and Methods

### 2.1. Materials

PGS pre-polymers (pPGS), sebacic acid powder (Sigma Aldrich, Seoul, Korea), and glycerol (>99.5%, Sigma Aldrich, Seoul, Korea) were prepared according to the previously published method [58]. A 1:1.5 molar mixture of sebacic acid and glycerol was placed in a three-neck round bottom flask. The monomers were heated at 120 °C in a nitrogen environment for 24 h. The synthesized solution was filtered using cold acetone (Sigma Aldrich, Seoul, Korea) to remove the loosely cross-linked monomers and the filtrate was used for experiments. The filtrate was reheated and a few drops of *N*,*N*-dimethyl formamide (DMF) solvent (Sigma Aldrich, Seoul, Korea) were added to make a homogeneous solution. The PVA solution was prepared by dissolving 1.5 g of PVA powder (Mw~27,000, Sigma Aldrich) in 10 mL of distilled water at 90 °C under continuous stirring for 4 h. Both solutions were blended at various loading ratios of PGS and PVA (2:1.5, 2:1, 2:2 (*v*/*v*)) at 50 °C for 30 min. The solution was processed for wet spinning, in which the coagulation bath contained acetone (Sigma Aldrich, Seoul, Korea) and water with an acetone concentration of 70% (*v*/*v*). The AuNPs (20 nm in diameter, Sigma Aldrich, Seoul, Korea) were incorporated in a composite conductive microfiber solution.

### 2.2. Fabrication of Microfiber

The composite solutions of PGS and 15 wt % of PVA at various loading ratios of PGS and PVA (2:1.5, 2:1, 2:2 (*v*/*v*)) were measured in a 3-mL plastic syringe. A 21-gauge stainless-steel needle was used for wet spinning. The process was conducted at an extrusion speed of 35 mL/h from the plastic syringe. The coagulation bath contained the acetone and water with a volume ratio of 70% (*v*/*v*) and extruded microfiber was left in the coagulation bath for 2 min after obtaining the required length. Microfibers were left to dry completely at room temperature. Mechanically strong microfibers with a diameter of about 350 µm were fabricated.

### 2.3. Cytotoxicity Test

The cytotoxicity of PGS:PVA composite microfiber was tested following the standard ISO10993-5 “Biological evaluation of medical devices “ tests for in vitro cytotoxicity: Indirect MTT (3-[4,5-dimethylthiazol-2-yl]-2,5-diphenyltetrazolium bromide) cytotoxicity tests were performed using the L929 mouse fibroblast cell line. The microfiber samples were sterilized by UV light to prevent bacterial contamination. L929 mouse fibroblasts were used to determine the biocompatibility and cytotoxicity of the composite microfibers. The cells were cultured in minimum essential medium (MEM) supplemented with 10% fetal bovine serum and 2% penicillin/streptomycin at 37 °C in a humidified atmosphere of 5% carbon dioxide and 95% air. L929 fibroblasts were seeded in 12-well plates in a 1 mL culture medium at a concentration of 1 × 10^5^ cells/mL and cultured for 24 h at 37 °C to 80% confluence. Then, the growth medium was removed, and microfiber samples were added carefully into each well. The extract of high-density polyethylene (HDPE) was added for the negative control and 10% dimethyl sulfoxide (DMSO) for the positive control. After 5 d of incubation at 37 °C, the cytotoxic effect was determined by observation using an inverted phase-contrast microscope. Quantitative evaluation of cytotoxicity was conducted by MTT assay, which is an assay of metabolism by mitochondrial dehydrogenase of active cells into formazan crystals. The L929 fibroblasts were seeded in 96-well plates at a density of 2 × 10^3^ cells per well. The cells were incubated for 24 h at 37 °C to 80% confluence. After 24 h, the culture medium was removed from the wells, and microfiber samples were added. After every 5 d of incubation, an MTT assay was performed to evaluate cell viability. A solution of 50 μL MTT (3-[4,5-dimethylthiazol-2-yl]-2,5-diphenyltetrazolium bromide) in phosphate-buffered saline solution was added to each well. After a further incubation period of 4 h at 37 °C, the supernatant was aspirated, and formazan crystals were dissolved by 150 μL DMSO. The absorbance was measured at 540 nm using a hybrid microplate reader (VersaMax; Molecular Devices, Sunnyvale, CA, USA). Cell viabilities were presented as the ratio of the absorbance of treated cells to the absorbance of control cells cultured with an extract of HDPE.

### 2.4. Fabrication of Microfiber-Based Strain Sensors

AuNPs (20 nm in diameter) were incorporated into solution of biodegradable-composite-microfiber and stirred for 30 min. The Au-incorporated microfibers were extruded at 35 mL/h from the 3 mL syringe in the coagulation bath and left for 1 min in the same bath after cutting it to the required length. The microfiber was dried at room temperature. The AuNPs-incorporated microfiber samples were stored in a desiccator for 1 day for further testing.

### 2.5. Characterization

Morphologies of the microfibers were evaluated by field-emission scanning electron microscopy (FE-SEM, JEOL JSM6500F, Tokyo, Japan). A degradation pattern was observed for the microfiber with a 2:1.5 ratio of PGS:PVA with Fluoresbrite^®^ dye (red) and the PVA microfiber with fluorescein isothiocyanate (FITC) dye (green) using fluorescence imaging with a confocal microscope (FLUOVIEW 3000, Olympus, Seoul, Korea). An ultimate tensile tester (LR10k-Plus, Shanghai, China) was used to measure the stress–strain behaviors of the biodegradable microfiber. The strain sensor response was measured in a custom-built stretching-bending system with a semiconductor parameter analyzer (4200A-SCS, Keithly, Seoul, Korea). The cell viability was measured using a microplate reader (Biotek Synergy H1, Hybrid, Seoul, Korea) and statistical software.

## 3. Results and Discussion

**Figure 1:**
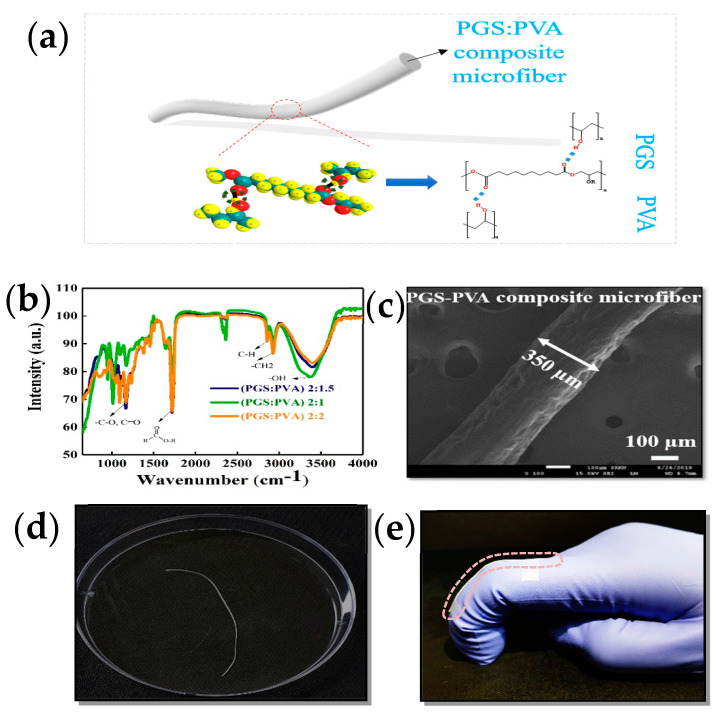
Schematic and characteristics of biodegradable microfiber. (**a**) Representation of PGS:PVA composite microfiber. Hydrogen bonding is shown by the dotted lines between the PGS and PVA in the composite. (**b**) FT-IR spectra of the prepared composite solutions at various ratios of PGS:PVA (2:1, 2:1.5 and 2:2 (*v*/*v*)). (**c**) A cross-sectional FE-SEM image of a microfiber with a diameter of about 350 µm. The scale bar is 10 µm. (**d**) The photograph shows the uniform length of the fabricated microfibers. (**e**) The photograph demonstrates the conformity of the microfiber to a human finger.

Due to the low viscous nature of PGS, additional physical or chemical processes are required to improve the viscosity and strength of PGS. PVA was blended with PGS to facilitate microfiber formation at PGS:PVA mass ratios of 2:1, 2:1.5, and 2:2. Biodegradable and stretchable composite microfibers of PVA and PGS were prepared in three steps (Figure 1, Supplementary Figure S1). In the first step, pre-polymer PGS (pPGS) was prepared according to a previously published report [58]. Briefly, a 1:1.5 molar mixture of glycerol and sebacic acid was heated at 120 °C under a nitrogen atmosphere for 24 h. In the second step, an aqueous solution of 15 wt % PVA was prepared by dissolving a weighed amount of PVA powder in distilled water at 90 °C with rapid stirring for 4 h. The weighed amount of PVA was prepared by adding a few drops of DMF in the third step, and the two solutions were blended to attain various mass ratios of PGS to PVA (2:1.5, 2:1, 2:2). The process sequence of composite solutions is shown schematically in Supplementary Figure S1.

The prepared composite solutions of PGS:PVA (2:1.5, 2:1, 2:2 in mass ratio) were placed in a 3 mL plastic syringe with a blunt 21-gauge stainless-steel needle for the wet-spinning experiment. The PGS-PVA composite microfibers were prepared by the conventional wet-spinning method. The schematic procedure for wet spinning is shown in Supplementary Figure S2. The wet-spinning process was performed by extrusion of composite solution at a speed of 35 mL/h from a 3 mL plastic syringe. Deionized (DI) water and acetone were mixed at a ratio of 7:3 (70% *v*/*v*) as a coagulation bath. The wet-spun microfiber was left in the coagulation bath for 2 min once the required length of composite microfiber was obtained. After wet-spinning, the microfibers were dried at room temperature for 3 h. The schematic of microfiber and hydrogen bonding between PGS and PVA is shown in Figure 1a and Supplementary Figure S3.

The microstructure of microfibers was evaluated using Fourier-transform infrared (FT-IR) spectroscopy and field-emission scanning electron microscopy (FE-SEM). The FT-IR spectra of wet-spun composite microfibers at various PGS:PVA ratios are shown in Figure 1b. The synthesized PGS exhibited a peak at 936 cm^−1^ which is correlated with an aliphatic acid of sebacic acid. The peak at 1733 cm^−1^ is correlated with carbonyl (C=O) groups in which an ester bond exists between glycerol and sebacic acid. The two shoulder peaks at 2854 and 2926 cm^−1^ are ascribed to methylene (–CH_2_) groups. The broad band from 3100 to 3500 cm^−1^ is related to O–H stretching, which shows homogeneous mixing of the composite solution. These bonds also indicate successful synthesis of PGS. Figure 1c displays the FE-SEM image of PGS-PVA microfiber with a selected ratio of 2:1.5 (PGS:PVA). The uniform and smooth surface morphology of the microfiber was observed. The diameter of composite microfiber was about 350 µm. An optical image of the composite microfiber is shown in Figure 1d. The uniformity of the 3.5-cm-long stretchable microfiber was confirmed by wet-spinning. The optical image of the microfiber with a mass ratio of 2:1.5 shows the conformality of the microfiber attached to a human finger while bending (Figure 1e).

**Figure 2:**
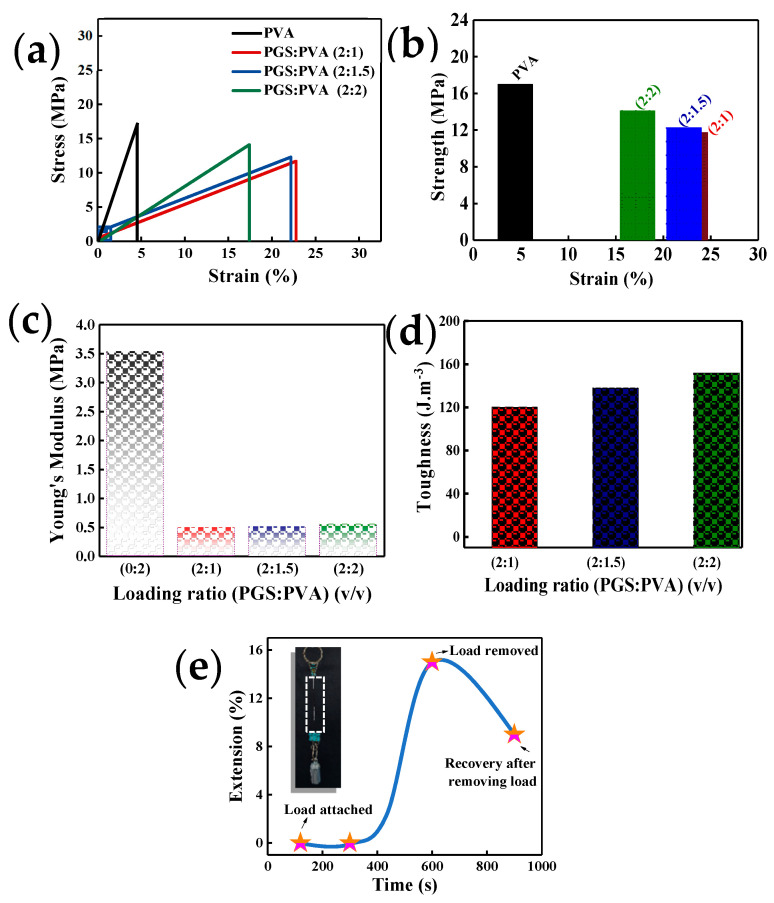
Mechanical properties of the biodegradable microfiber. (**a**) Stress-strain curves of the microfibers with varying loading ratios of PGS:PVA (2:1, 2:1.5, 2:2 (*v*/*v*)). (**b**) Strength, (**c**) Young’s modulus, and (**d**) toughness of the biodegradable microfibers with various loading ratios of PGS:PVA (2:1, 2:1.5, 2:2 (*v*/*v*)). (**e**) Creep test of a microfiber at a loading ratio of 2:1.5 under a hanging weight of 30 g at room temperature. The initial length is measured before addition of the hanging weight, and the final length change is in terms of extension% measured after removal of the weight.

For mechanical characterization of composite microfibers, a uniaxial tensile test was performed using a load cell of 100 N. Samples were cut to a rectangular shape (5 cm × 2 cm), and the crosshead speed was set to 2 mm/min during the uniaxial tensile test. Young’s modulus was calculated in the linear range of the stress-strain curve. The ultimate tensile strength was measured as the highest peak of the stress-strain curve. Figure 2a shows the typical stress-strain curves of the composite microfiber at various PGS:PVA ratios. Linear tensile behavior in Figure 2a revealed that PGS with low Young’s modulus imparts stretchability, and brittle PVA with high Young’s modulus imparts strength and stability to the composite microfiber at various ratios of PGS:PVA. The high Young’s modulus of pristine PVA shows a high strength of 18 MPa at 4% strain. The composite microfiber shows strain at break values of 23%, 22%, and 18% and tensile strengths of 11 MPa, 13 MPa, and 16 MPa for PGS:PVA mass ratios of 2:1, 2:1.5, and 2:2, respectively (Figure 2b). When applied to the body, the Young’s modulus of the composite microfiber matches that of human skin, which could minimize the need for geometric compensation at different stiffness. Young’s moduli of composite microfibers were 0.5, 0.6, and 0.65 MPa at PGS:PVA ratios of 2:1, 2:1.5, and 2:2, respectively, as shown in Figure 2c, which matches the modulus range of human tissues. The Young’s modulus of the human lower back, human finger skin, and adipose tissues in the esophagus are 0.54, 0.4, and 0.45 MPa, respectively [59,60,61,62]. The PGS:PVA composite offers the possibility of tailoring mechanical properties by adjusting the mass ratio. The toughness, as shown in Figure 2d, increases with PVA content.

The formula for measuring Young’s modulus is

E = σ/ε(1)

where

σ = stress (MPa)(2)

ε = strain

E = Young’s modulus of elasticity

Young’s modulus is also known as the elastic modulus, and it is the ratio between the stress and strain.

A strain is defined as change in the dimension of materials and any force that act on material to produce stress. In the case of elastic tensile material, the strain is similar to stretch and it is the ratio of change in size to some basic size, often mentioned in terms percentage. Stress is force per unit area and the unit for stress is Pascal (Pa) or megapascal (MPa).

Furthermore, the stability of the mechanical properties of the composite microfiber was investigated by measuring hysteresis characteristics in the stress-strain curves (Supplementary Figure S4). Hysteresis is the energy loss during a given cycle of deformation and energy. When stress is applied to the composite microfiber, the long chain of polymer composites experiences position changes, and this rearrangement results in the observed hysteresis. When stress is applied to the microfiber, there is a back stress created of the same magnitude. When the applied stress is removed, the accumulated back stress causes the microfiber to return to its original form. The stress–strain hysteresis curves from the sample with a PGS:PVA loading ratio of 2:1.5 show recovery of the composite microfiber after each cycle of loading and unloading. The data in Supplementary Figure S4 show that the composite microfiber returns to its original shape after three cycles.

In human skin, hysteresis behavior is due mainly to the extracellular matrix of the dermis, where fine elastin fibers provide elastic recovery and skin extensibility at relatively low strain. Stretchable PGS is the main component of recovery from hysteresis. The creep test was performed to investigate the elastic response of composite microfiber with a PGS:PVA loading ratio of 2:1.5 at a hanging weight of 30 g (Figure 2e). Mechanical creep is an acute response to stretching where the viscoelasticity of human skin allows it to be deformed in response to a loading or stretching force. For this test, a 30 g weight was hung at the end of the composite microfiber. The microfiber with the hanging load did not show any extension until 100 and 300 s, which means that the microfiber remained resistant to the weight until 300 s. The microfiber with a hanging load of 30 N was elongated at 600 s, after which it was extended by 15% from its original position. When the load was removed at 900 s, the microfiber recovered to an extension of 9%, which indicates creep. The data in Figure 2e indicate that creep was produced in the sample, but the microfiber could resist the creep for a specific duration.

**Figure 3:**
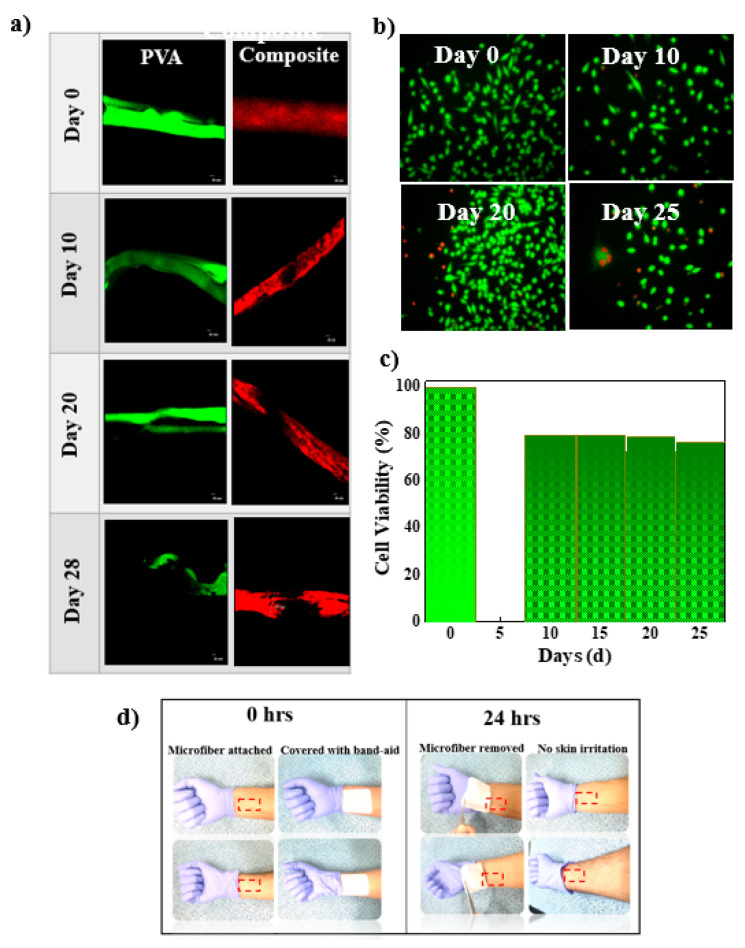
Biodegradability of the microfiber. The PVA microfiber and biodegradable PGS:PVA composite microfiber with a loading ratio of 2:1.5 were modified by incorporating FITC and Fluoresbrite^®^dye, respectively, and the images were captured using a confocal microscope. (**a**) Biodegradability pattern of PVA (green) and PGS:PVA (red) microfibers from 0 to 28 days. The composite microfiber shows the fast degradation while stiff PVA shows slow degradation. After 28 days most of the the PVA and composite microfibers are degraded at set conditions. (**b**) Cell viability was assayed by LIVE/DEAD cell staining in the solution (live cells as green fluorescence and dead cells as red fluorescence) at an interval of 5 days. The scale bar is 20 µm. (**c**) Cell viability data evaluated by LIVE/DEAD cell staining. (**d**) Photographs of a 1cm long microfiber attached to skin with a cotton band-aid (left panel). After 24 h, the sample was removed (right panel) from the skin, and no redness or irritation was observed. After removing the microfiber there was no allergic reaction and no itching was reported by any of the four volunteers.

The degradation behaviors of two samples were observed (PVA microfiber and composite PGS:PVA microfiber) by using an in-situ confocal microscopy. PGS has low formability and can lose its shape and so it is impossible to form the microfiber only with PGS. To differentiate between both samples, two colors of dyes were chosen. The green-fluorescent dye was designated to PVA microfiber and the red-fluorescent dye was designated to PGS: PVA (2:1.5 loading ratio). The degradation behaviors of the PVA microfiber and composite microfiber were evaluated in phosphate-buffered saline (PBS) solution under the static condition at 37 °C for 30 d. The samples with a length of 1 cm were placed in a small petri dish with a low evaporation lid in a humid atmosphere, and 4 mL of PBS was added to cover the samples completely. The dishes were incubated at 37 °C under humid conditions. The samples were washed with DI water and left to dry for 1 h. PVA with green-fluorescence dye optically showed slow degradation from day 0 to day 20 due to its stiff nature and high Young’s modulus. The dye was apparently bled into PBS solution after the PVA lost its par and degraded in the same PBS. The part of PVA-microfiber that was degraded appeared as a dark (black) area in the confocal imaging (circled with yellow line). Meanwhile, PVA microfiber lost its strength from day 0 to day 28, which made its handling difficult. The process was repeated every 5 d, and confocal images were captured to monitor the degradation of the microfiber (Figure 3a and Supplementary Figure S5). The PGS:PVA microfiber demonstrated fast degradation from day 0 to day 28 due to low strength of composite microfiber while the PVA microfiber showed slow degradation. After 28 d, most of the PVA microfiber and PGS:PVA composite microfiber was degraded in PBS solution. PGS degrades primarily through hydrolysis of the ester linkage into smaller oligomers and, ultimately, to the starting monomers, glycerol and sebacic acid. PGS degradation is unique and differs from that of other resorbable polymers (e.g., polylactide, polyglycolide, and copolymers) in that it degrades via surface erosion as opposed to bulk erosion. The weight loss% was not quantified. This can be checked by following the same procedure for next 28 days. The thick sheet of PGS:PVA with high content of PVA and varying temperature and time was degraded in PBS in 60 days [63]. The hydrolytic degradation of PGS into its component monomers, glycerol, and sebacic acid, provides a resorbable material with high biocompatibility. In the composite microfiber of PGS and PVA, surface degradation occurs when PBS starts to penetrate into the microfiber, but this occurs slowly due to PVA. The results illustrate the slow but continuous degradation of PVA and PVA:PGS microfibers over 28 d.

Based on the results of an indirect cytotoxicity test, the behaviors of fibroblast cells are shown in Figure 3b and Supplementary Figure S5. The PGS: PVA (2:1.5) microfiber samples were dipped into the cell media and removed every 5 d, and the cells were observed under fluorescent microscopy. The cells remained alive until 25 d, and no cytotoxicity was observed in an indirect test. The viability assay (MTT assay) showed that the composite microfiber had cell viability <78% without significant toxicity (Figure 3c) due to the nontoxic monomers of PGS and the biocompatible nature of PVA. Dermal fibroblast cells were alive even after 25 d, indicating that the materials promote cell biocompatibility. It is highly expected that cells may survive till 28 days (three days more than our performed experiment) as the PGS degrades into the monomers of glycerol and sebacic acid that are highly compatible. Glycerol has been used in pharmaceuticals and building blocks of lipids. Intermediate in the ω- oxidation of long and medium-chain fatty acids, the sebacic acid is the natural metabolite. Further, copolymers containing sebacic acid have been used in pharmaceutical drug delivery [63,64]. Poly (vinyl alcohol) (PVA) is also a well-established, FDA-approved polymer that is biocompatible, non-toxic, degradable, and has been used for in vivo application and in vitro green electronics [65,66]. Poly (vinyl alcohol) (PVA) is a well-known synthetic biocompatible polymer suitable for a range of pharmaceutical uses, so it can be used as a matrix for the incorporation of functional materials and has a wide range of applications in the cosmetics, food, pharmaceutical, and packaging industries. It was reported that the cell viability of PVA is almost 98% [67,68].

For wearable applications, biocompatibility without an allergic reaction, irritation (which can vary from person to person), or rash is required. The 1-cm-long microfibers with PGS:PVA loading ratio of 2:1.5 were attached to human skin on the arms of four volunteers with a cotton band-aid for 24 h. After 24 h, the cotton band-aid was removed, and no rash, itching, or irritation was observed. The pictures of skin area after attaching the microfiber are shown in Figure 3d (left panel). When the microfiber was removed from the skin after 24 h, no inflammation or any kind of reaction on the human arm was observed (Figure 3d (right panel) and Supplementary Figure S6). The above results of the indirect cytotoxicity test with L929 cells and skin-irritation test demonstrate the biocompatibility of PGS:PVA composite microfiber, suggesting this microfiber as a suitable candidate for potential wearable applications.

**Figure 4:**
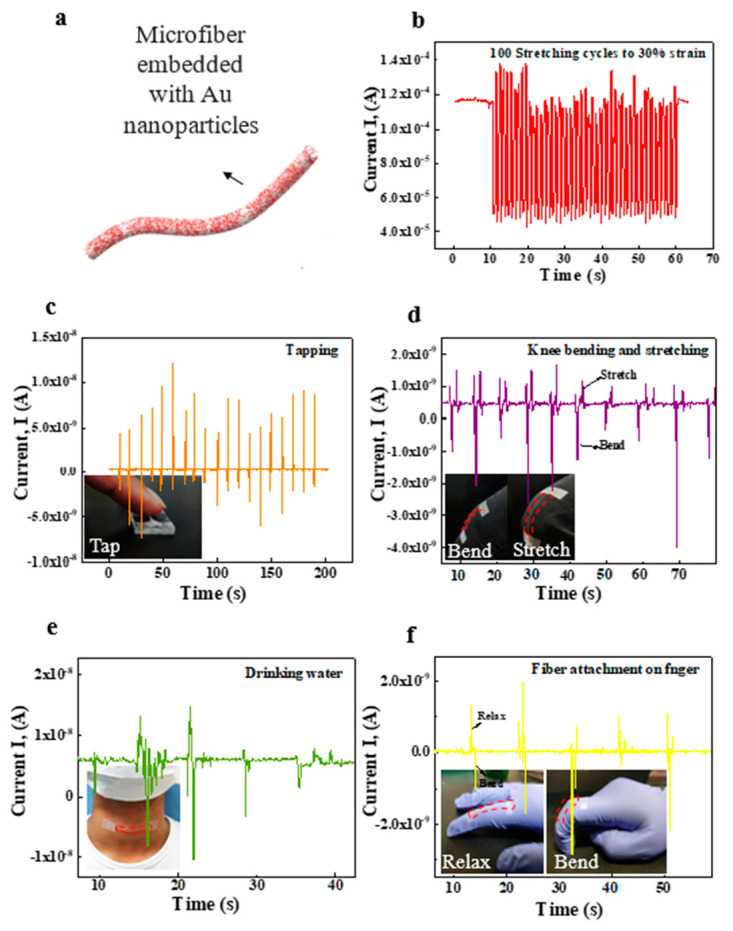
Demonstration of the biodegradable and stretchable strain sensor. (**a**) Schematic of AuNPs incorporated into PGS:PVA microfiber. (**b**) The current variation during cyclic stretching (100 stretch-release cycles) at 30% strain. (**c**) The current variation was recorded by the AuNP- incorporated strain sensor during tapping of the finger. The inset photograph shows the pressure applied by the finger on the strain sensor. (**d**) Response of the biodegradable strain sensor to bending and stretching of the knee. The inset is a photograph of the strain sensor attached conformally to the knee during bending and stretching. (**e**) The wearable strain sensor was attached to the esophagus, and current-time (I-T) response was measured during the movements. Inset photograph shows the strain sensor attached to the esophagus. (**f**) Response (I-T) of the wearable strain sensor to bending/relaxing of finger joints. The inset shows a photograph of microfiber-based stretchable strain sensors attached to the finger joints.

Transient wearable electronics usually consists of a thin layer of stiff materials deposited onto a stretchable substrate. When the substrate is stretched, the thin layer of stiff material is deformed, resulting in cracking and delamination of the layer. Therefore, the failure of electronics when subjected to mechanical strain represents a challenge in transient stretchable electronics. We investigated the mechanical and electrical stability of conductive stretchable PGS:PVA composite microfiber embedded with AuNPs through static and cyclic stretching tests. For that purpose, AuNPs (0.5 g) were added to the PGS:PVA solution (2:1.5) by continuous stirring for 30 min. The conductive microfiber with a length of 3 cm was fabricated by wet spinning (Figure 4a). To investigate the responsivity of the AuNP-incorporated microfiber to strain, the current was measured. The results in Supplementary Figure S7 show the current-voltage (I-V) curves for the biodegradable microfiber embedded with 0.5 g of AuNPs. The current is in the range of μA and shows that the AuNPs enabled the formation of a conductive path in the microfiber.

The electrical resistance change, ΔR/R_0_ = ((R − R_0_)/R_0_) × 100%, where R_0_ is the initial resistance and R is the resistance measured after a certain amount of strain, of the AuNP-incorporated microfiber was measured during static stretching. To investigate the ΔR/R_0_ change of AuNP-incorporated microfiber due to mechanical deformation, the microfiber samples were subjected to static stretching at strains of 0%, 10%, 20%, and 30%. The ΔR/R_0_ value of the conductive microfiber increased significantly from 0% to 30% at a static stretched condition due to an increase in disconnection of electrical conduction in the microfiber with an increase in stretching strain. Above 30% strain, the ΔR/R_0_ value sharply increased due to the complete loss of electrical conduction in the microfiber (Supplementary Figure S7).

The current-time (I-T) curves at a bias of 0.5 V were obtained after cyclic stretching and are presented as the current I versus time after a certain number of stretching cycles with 10%, 20%, and 30% strain (Supplementary Figure S8). As the amplitude of applied strain increased from 10% to 30%, the I value started to decrease. The I value also decreased with increasing stretching cycles (0–4000) at strains of 10%, 20%, and 30%, which shows the stability and repetitive response of the conductive microfiber up to 30% stretching. To investigate the electrical property of the strain sensor under dynamic stretching, the time-dependent real-time current was measured at 10% strain (20 and 50 cycles) and 30% strain (20 and 50 cycles) (Supplementary Figures S9 and S10, respectively). The results of I-T showed variations in the response during dynamic cyclic stretching. The observed current variation under dynamic stretching of the microfiber (20 and 50 cycles) indicates the effective response of AuNPs in composite microfiber.

The results in Figure 4 indicate the high sensitivity and good repeatability of the AuNP-incorporated microfiber-based strain sensor. The biodegradable and stretchable strain sensor was able to distinguish between the various motions, showing a unique signal pattern for each. Our results showed that the sensor accurately measured the deformation produced by tapping on the microfiber, knee bending and stretching, swallowing, and finger bending/relaxing. Future applications can be expanded to sophisticated activity recognition in biomedical fields and detection for human–machine interfaces.

To demonstrate the potential application of the strain sensor of the biodegradable composite microfiber incorporated with AuNPs in detecting human motions (Figure 4a), the time-dependent current (I-T) was measured at 30% strain over 100 dynamic cycles (Figure 4b) to observe the behavior of the strain sensor under cyclic stretching. The variation in I shows the effective response of the sensor under 30% stretching. To determine the ability of the sensor to detect the strain, the microfiber was tapped with a finger, and the current variation with respect to time was observed (Figure 4c). The sensor was attached to the knee while the person sat with a naturally relaxed leg. On bending, the large strain was accommodated by the sensor, and there was a decrease in I. Then, the leg was stretched and the natural strain decreased, which increased I. This resistive-type stretchable sensor is beneficial for low-strain motions due to its high sensitivity. To determine the ability of the sensor to detect the motion of the knee stretching and bending, time-dependent I was measured (Figure 4d). The data showed that the detected motion was stable and periodic with high accuracy. Furthermore, a strain sensor was attached to the neck to detect muscle motion near the throat (Figure 4e). When the subject swallowed saliva, current changes of the sensor were detected. Similar current variations were produced when the subject swallowed saliva repeatedly, indicating the high sensitivity and stability of the sensor. The strain sensor was also attached to the finger of a glove to measure the electrical response at the bent and relaxed positions (Figure 4f). When a finger joint was bent, the bending strain was accommodated by the strain sensors, causing a change in the electrical signal. When the finger was straightened, the strain sensor was relaxed, accompanied by recovery of the electrical current. This biodegradable and stretchable strain sensor can monitor human motions, which is highly desirable for the development of transient wearable electronic devices and healthcare monitoring systems.

## 4. Conclusions

We proposed and fabricated a biodegradable, stretchable, and mechanically stable microfiber. The composite microfiber was formed of low-strength PGS elastomer and high-strength PVA using wet spinning to overcome the drawbacks of PGS, such as low viscosity, processability, and formability. The microfiber exhibited mechanical stretchability and stability due to the use of PGS elastomer-based composite. The biodegradation study showed the degradation of microfiber over 28 d. Characterization of the microfiber using skin-irritation tests and cell viability measurements indicated high biocompatibility. The AuNP-incorporated conductive microfibers showed repeatability of the electrical response under cyclic (up to 4000 cycles at 10%, 20%, and 30% strain) and dynamic stretching (at 10% and 30% strain), indicating the potential for applications of composite-based biodegradable and stretchable microfibers in stretchable devices. The microfiber-based strain sensor attached to the human body demonstrated the capability of detecting minute strains on the sensor induced by bodily activities. In summary, we improved the mechanical properties and formability of viscous PGS by adding stiff PVA. This is the first study utilizing wet-spinning to fabricate a PGS:PVA composite microfiber and microfiber-based strain sensor. The principle of making biodegradable and stretchable composite materials based on PGS elastomer has the potential to be extended to fabricate other composites with biodegradable and stretchable on-body wearable or in-body implantable applications.

## Data Availability

The data presented in this study are available in the article and Supplementary Materials.

## Supplementary Materials

The following are available online at https://www.mdpi.com/article/10.3390/mi12091036/s1, Figure S1: Preparation of PGS:PVA composite microfiber solution; Figure S2: Wet spinning process; Figure S3: Chemical scheme of PGS synthesis by polycondensation of glycerol and sebacic acid; Figure S4: The stress-strain hysteresis curves of microfiber at a PGS:PVA loading ratio of 2:1.5 (*v*/*v*); Figure S5: The PVA microfiber and PGS:PVA composite microfibers with a loading mass ratio of 2:1.5 were modified by incorporating fluorescein isothiocyanate (FITC) in PVA and Fluoresbrite^®^ dye into biodegradable microfiber, and the images were captured using a confocal microscope. (**a**) Biodegradability pattern of PVA (green) and PGS:PVA (red) microfiber at days 5, 15, and 25. (**b**) Cell viability was assayed by LIVE/DEAD cell staining in the solution of the control sample and of that at day 15 after the microfiber was dipped in the cell media. Green and red fluorescent cells indicate live and dead cells, respectively; Figure S6: Photographs of microfiber attached to skin of two volunteers with a cotton band-aid; Figure S7: Current-voltage (I-V) curves for the biodegradable microfiber embedded with 0.5 g of AuNPs; Figure S8: Current-time (I-T) curves of the cyclically stretched conductive microfiber; Figure S9: The time-dependent real-time current response; Figure S10: The time-dependent real-time current response. (**a**) 30% strain (20 cycles) and (**b**) 30% strain (50 cycles).
