# Supplementary material for: A Composite Microfiber for Biodegradable Stretchable Electronics
Source: Micromachines (Basel). 2021 Aug 28;12(9):1036. doi: 10.3390/mi12091036 (PMC8468109; doi:10.3390/mi12091036)
Supplement: Supplementary file 1 [file micromachines-12-01036-s001.zip › micromachines-1339229-supplementary.pdf]

micromachines

*Supplementary Material*

# A Composite Microfiber for Biodegradable Stretchable Electronics

**Adeela Hanif [1,†], Gargi Ghosh [1,†], Montri Meeseepong [2], Hamna Haq Chouhdry [2], Atanu Bag [1], M. V. Chinnamani [1], Surjeet Kumar [1], Muhammad Junaid Sultan [1], Anupama Yadav [1] and Nae-Eung Lee [1,2,3,*]**

1 School of Advanced Materials Science and Engineering, Sungkyunkwan University (SKKU), Suwon, Kyunggi-do 16419, Korea; adeelahaneef@gmail.com (A.H.); gargisimita@gmail.com (G.G.); atanubagec@gmail.com (A.B.); chinnabme@gmail.com (M.V.C.); surjeetkhurana9@gmail.com (S.K.); mjunaidsultan30@gmail.com (M.J.S.); anupama.yadav.biotech@gmail.com (A.Y.)
2 SKKU Advanced Institute of Nanotechnology (SAINT), Sungkyunkwan University (SKKU), Suwon, Kyunggi-do 16419, Korea; chivazh.mm@gmail.com (M.M.); Hamna.haq.ch@gmail.com (H.H.C.)
3 SKKU Advanced Institute of Nanotechnology (SAINT), Biomedical Institute for Convergence at SKKU (BICS), Sungkyunkwan University (SKKU), Suwon, Kyunggi-do 16419, Korea
* Correspondence: nelee@skku.edu
† These authors contributed equally to this work.

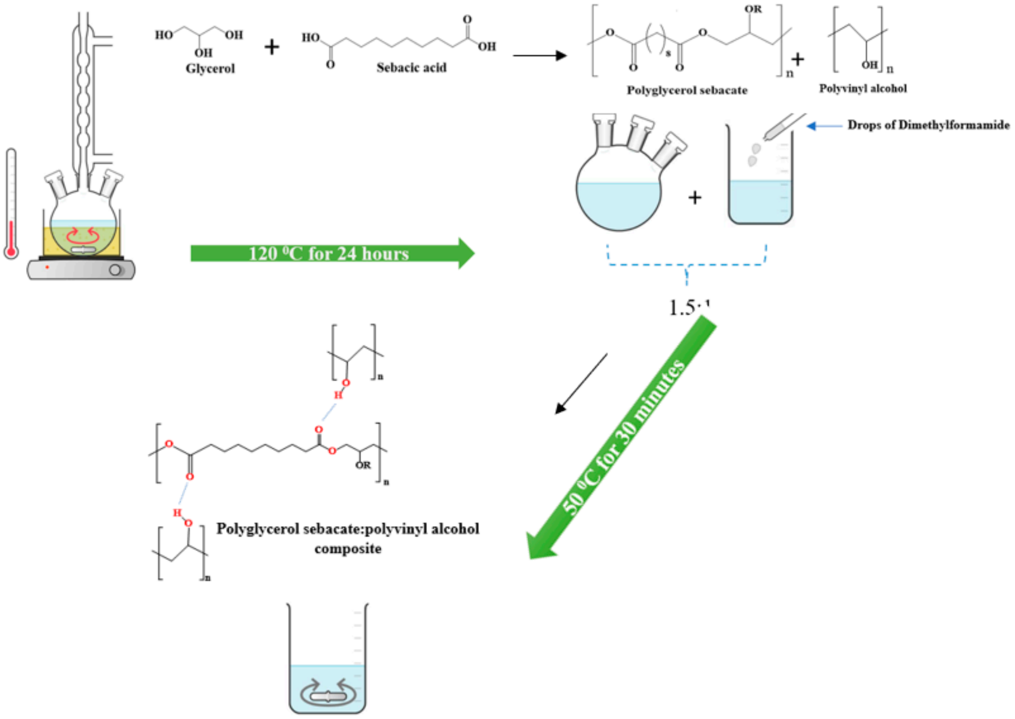


**Figure S1.** Preparation of PGS:PVA composite microfiber solution. PGS pre-polymers (pPGS) were prepared from glycerol and sebacic acid monomers. A 1:1.5 molar mixture of sebacic acid and glycerol was placed in a three-neck round bottom flask. The monomers were heated at 120 °C in a nitrogen environment for 24 h. The synthesized solution was filtered using cold acetone to remove the loosely cross-linked monomers, and the filtrate was used for our experiment. This filtrate was reheated, and a few drops of DMF solvent were added to make a homogeneous solution. The PVA solution was prepared by dissolving the 1.5 g of PVA powder in 10 mL of distilled water at 90 °C under continuous stirring for 4 h. The solutions were blended at various mass ratios of PGS and PVA at 50 °C for 30 min.

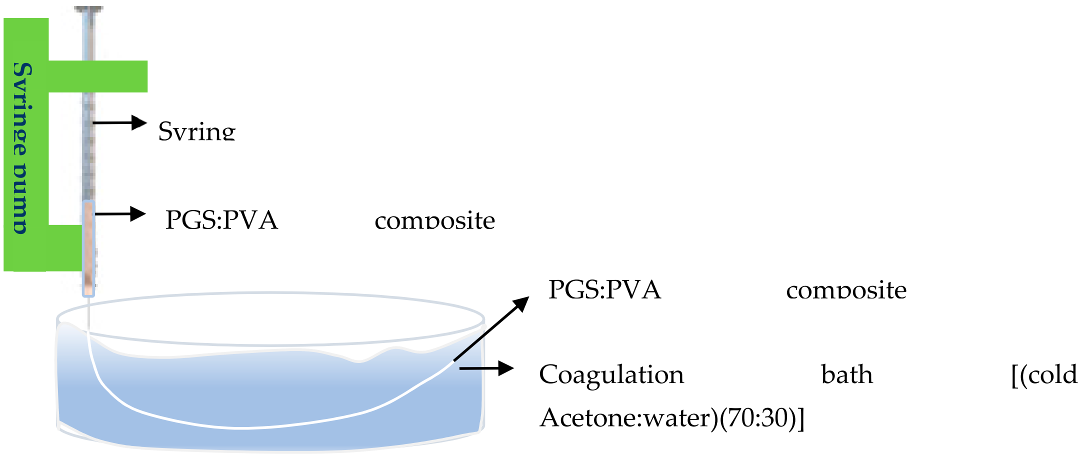


**Figure S2.** Wet spinning process. PGS:PVA solutions at various loading ratios (2:1.5, 2:1, 2:2) (v/v) were uptaken into a plastic syringe of 3 ml and extruded at a speed of 35 ml/hr. The coagulation bath contained acetone and water at a volume ratio of 70% (v/v), and extruded microfiber was left in the coagulation bath for 2 min after obtaining the required length. Microfibers were dried completely at room temperature.

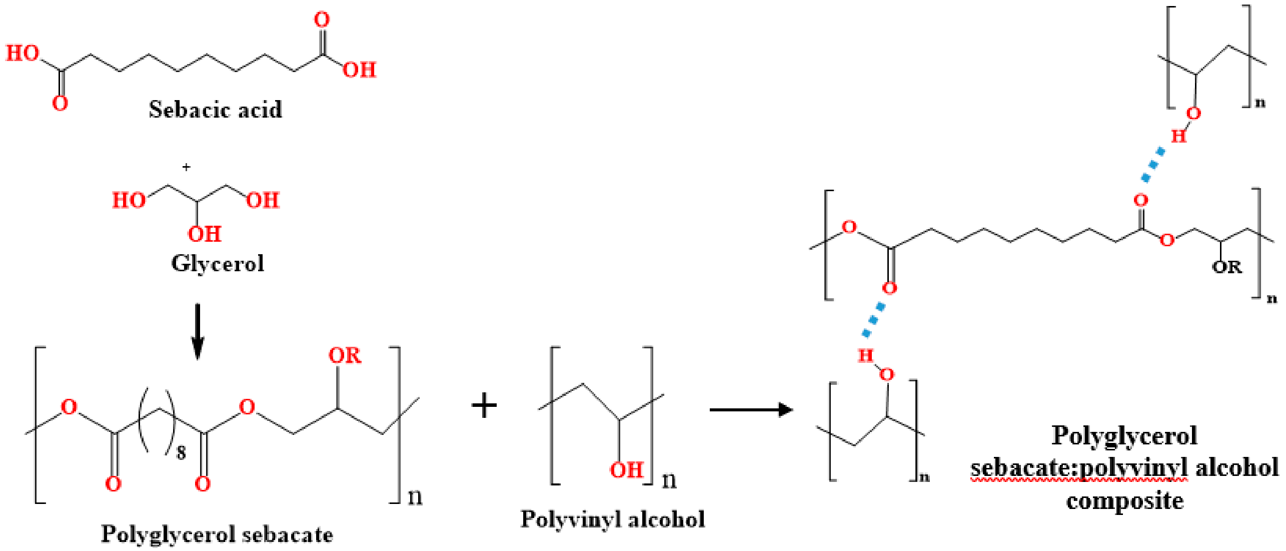


**Figure S3.** Chemical scheme of PGS synthesis by polycondensation of glycerol and sebacic acid. The composite solution was prepared by adding PVA. The scheme shows the hydrogen bond between PGS and PVA in the composite.

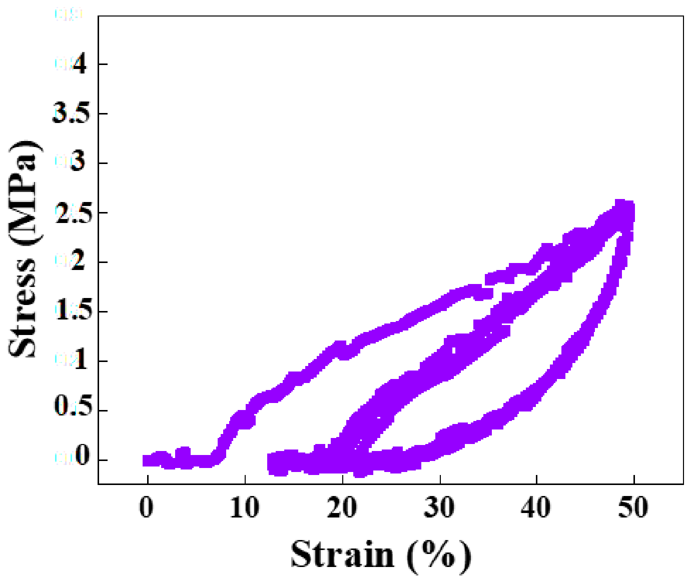


**Figure S4.** The stress-strain hysteresis curves of microfiber at a PGS:PVA loading ratio of 2:1.5 (v/v). The hysteresis characteristics are attributed to the viscoelastic characteristics of microfiber. Data were obtained for three cycles. .

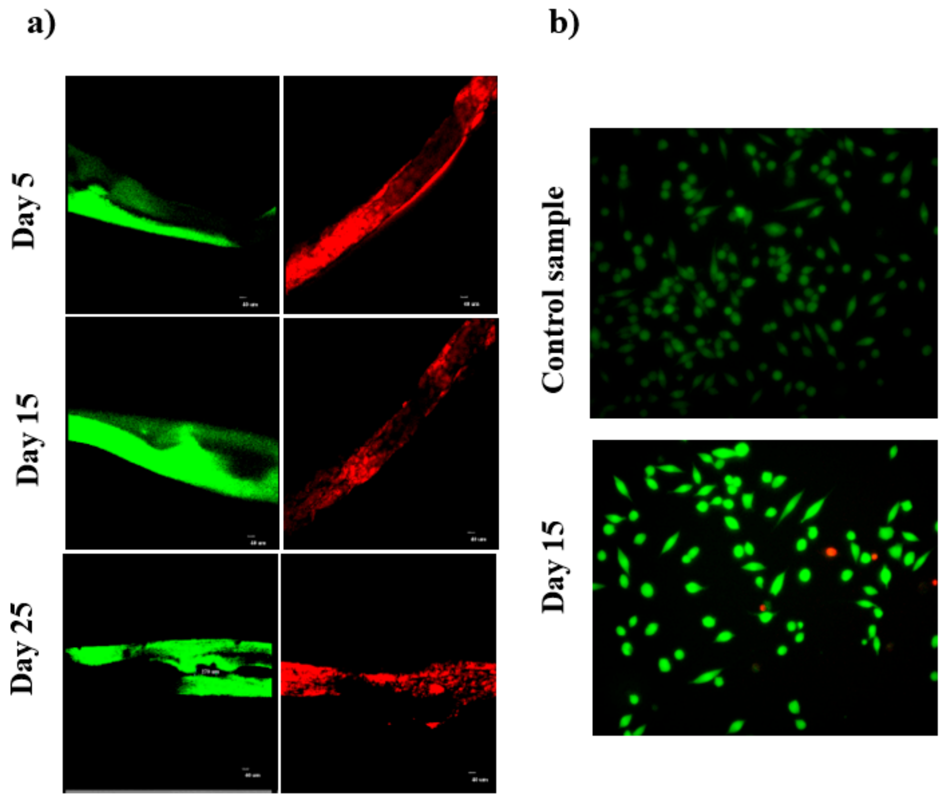


**Figure S5.** The PVA microfiber and PGS:PVA composite microfibers with a loading mass ratio of 2:1.5 were modified by incorporating fluorescein isothiocyanate (FITC) in PVA and Fluoresbrite®dye into biodegradable microfiber, and the images were captured using a confocal microscope. (**a**) Biodegradability pattern of PVA (green) and PGS:PVA (red) microfiber at days 5, 15, and 25. (**b**) Cell viability was assayed by LIVE/DEAD cell staining in the solution of the control sample and of that at day 15 after the microfiber was dipped in the cell media. Green and red fluorescent cells indicate live and dead cells, respectively.

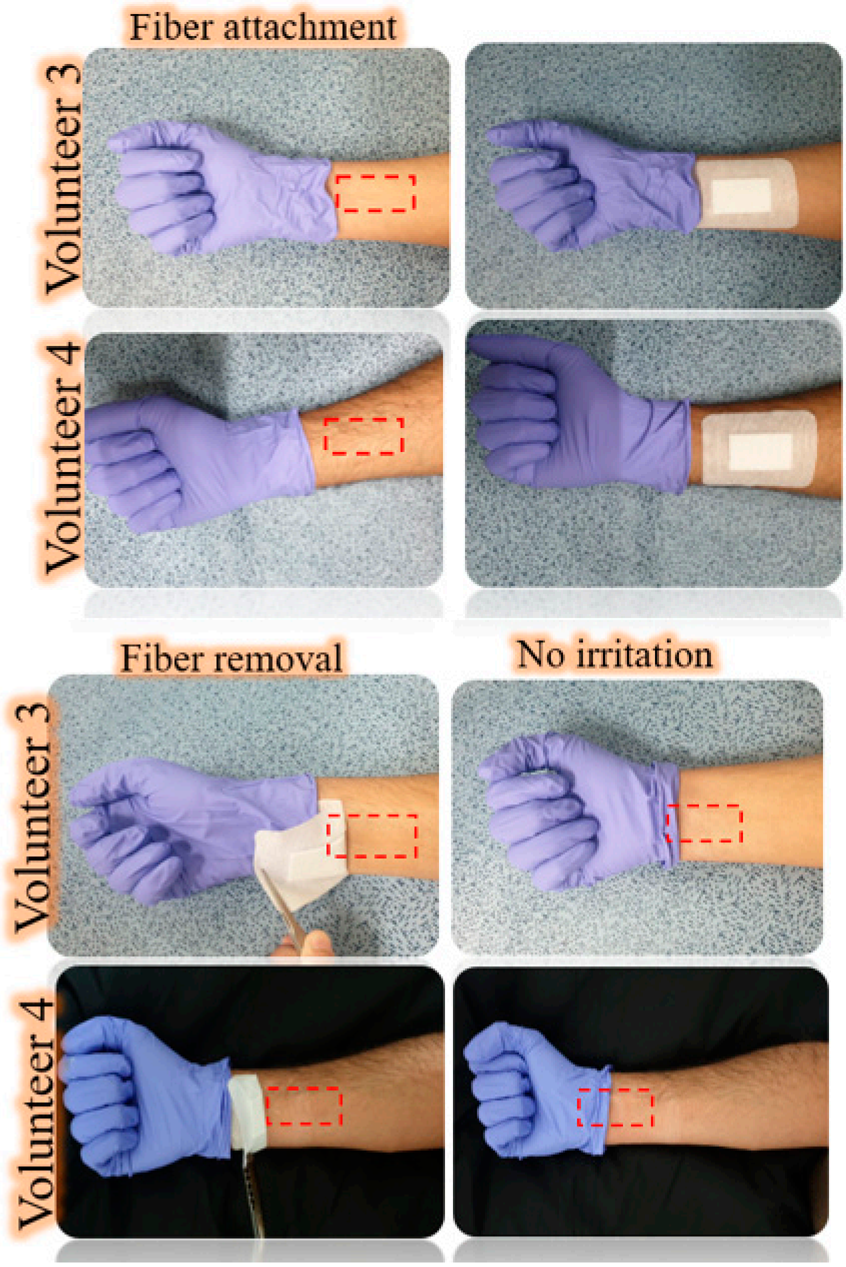


**Figure S6.** Photographs of microfiber attached to skin of two volunteers with a cotton band-aid. After 24 h, the sample was removed from the skin. No redness or irritation was observed.

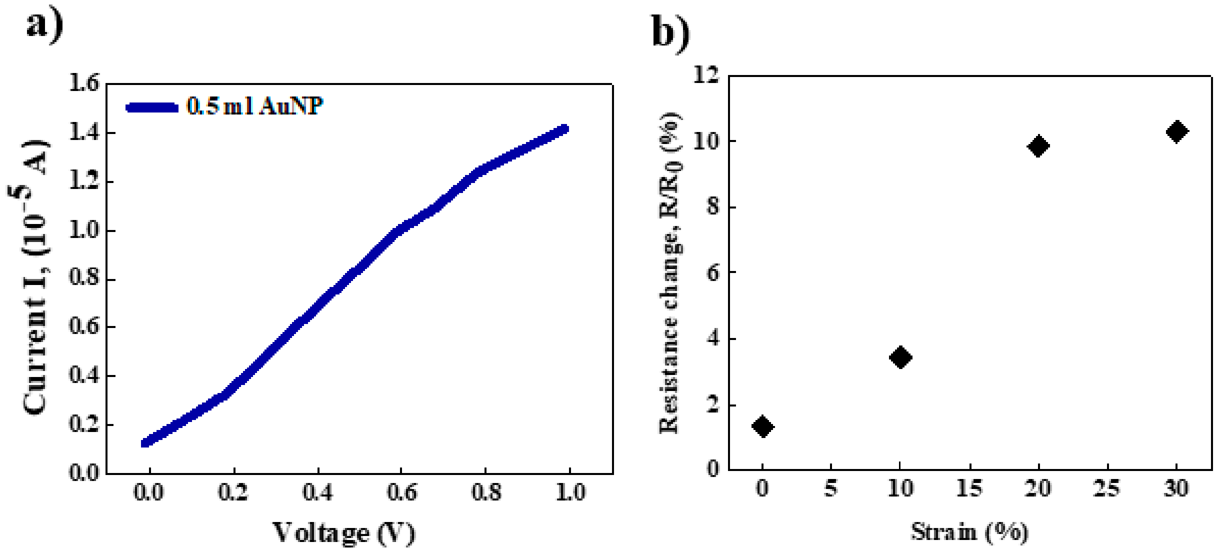


**Figure S7.** Current-voltage (I-V) curves for the biodegradable microfiber embedded with 0.5 g of AuNPs. The $\Delta R/R_0$ value of the conductive microfiber increased significantly from 0% to 30% at static stretched condition.

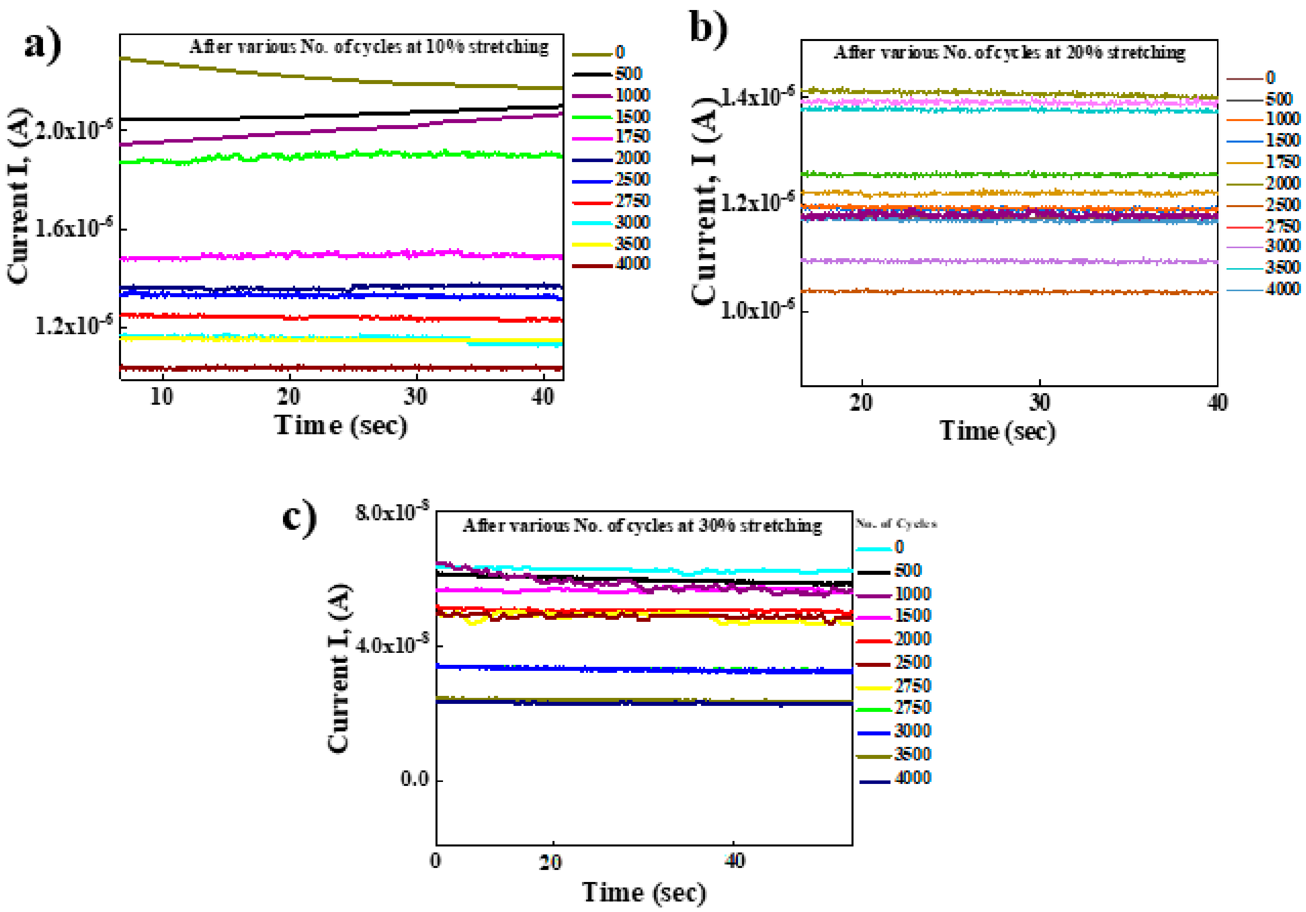


**Figure S8.** Current-time (I-T) curves of the cyclically stretched conductive microfiber. The current I versus time T at a bias of 0.5 V was obtained at elongation of (**a**) 10%, (**b**) 20%, and (**c**) 30% strain after cyclic stretching from 0 to 4,000 cycles.

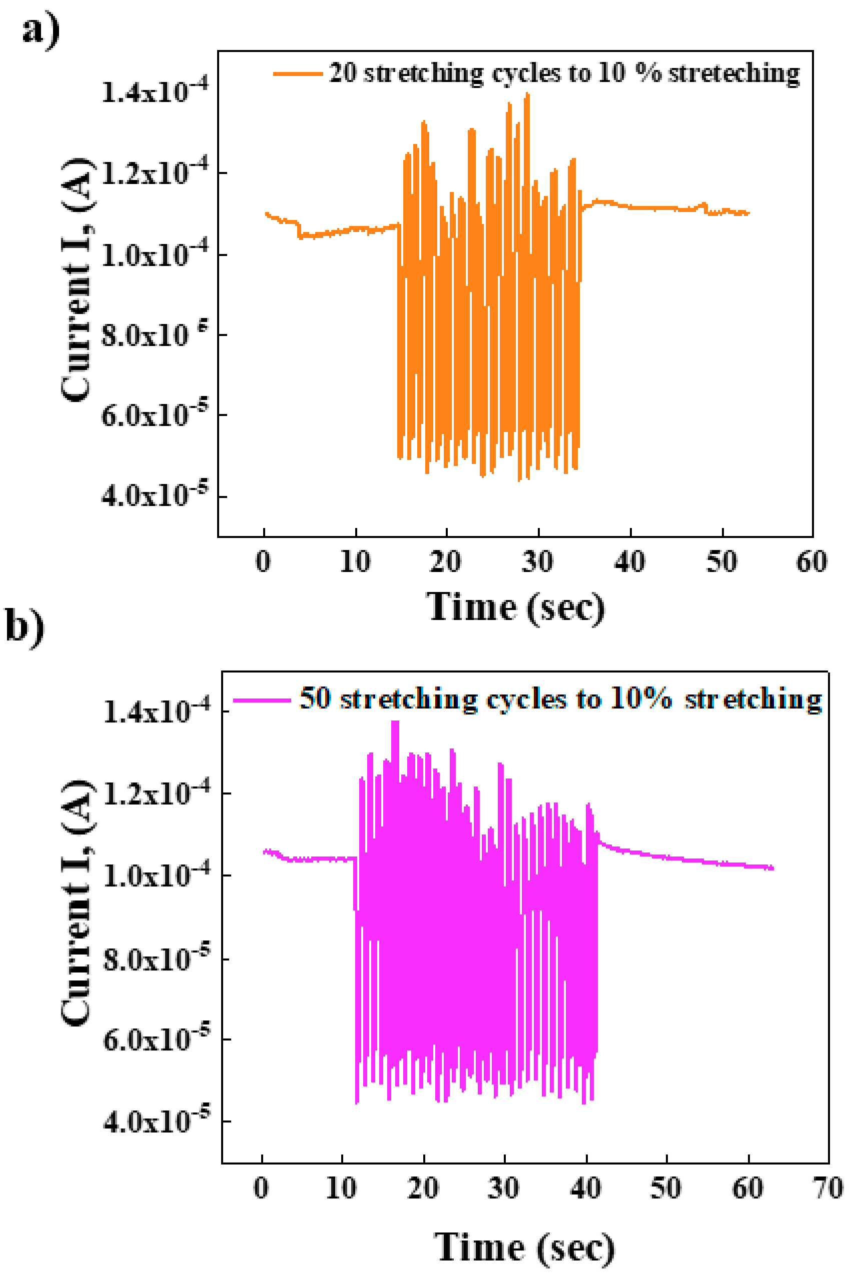


**Figure S9.** The time-dependent real-time current response. (**a**) 10% strain (20 cycles) and (**b**) 30% strain (50 cycles).

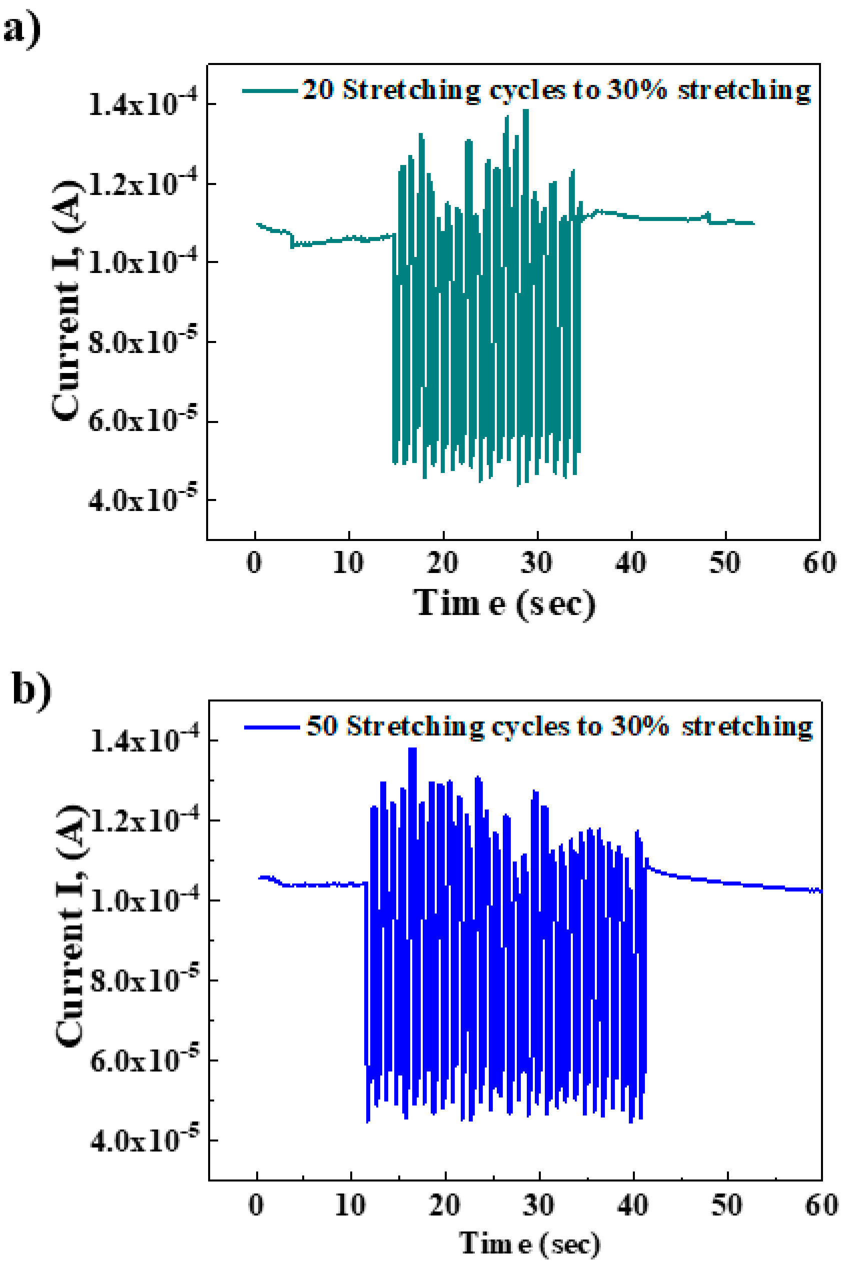


**Figure S10.** The time-dependent real-time current response. (**a**) 30% strain (20 cycles) and (**b**) 30% strain (50 cycles).
